# Metabolic contributions to hamstring muscle damage during maximal, high‐speed eccentric exercise in males

**DOI:** 10.14814/phy2.70441

**Published:** 2025-07-03

**Authors:** Carsten Schwiete, Joachim Mester, Patrick Wahl, Holger Broich, Michael Behringer

**Affiliations:** ^1^ Department of Sports Sciences Goethe University Frankfurt Frankfurt am Main Germany; ^2^ German Research Centre of Elite Sport German Sport University Cologne Cologne Germany; ^3^ Section Exercise Physiology German Sports University Cologne Cologne Germany; ^4^ Department of Performance, Neuroscience, Therapy, and Health Medical School Hamburg Hamburg Germany

**Keywords:** metabolism, muscle damage, muscle fatigue

## Abstract

High‐velocity eccentric training elicits exercise‐induced muscle damage (EIMD), predominantly attributed to mechanical strain. However, the potential contribution of metabolic stress to muscle damage remains underexplored, especially in trained populations. This study examined whether metabolic changes contribute to EIMD during maximal eccentric hamstring curls. Thirty male participants performed five sets of 15 maximal eccentric leg curls at 210°/s. Muscle oxygenation (SmO₂) and pulmonary gas exchange (VO_2_ and VCO₂) were recorded during the intervention. Creatine kinase (CK), muscle soreness, muscle stiffness, muscle contractility, peak torque, and maximal voluntary contraction (MVC) were measured pre‐exercise, post, and over 96 h of recovery. Linear mixed models were used to analyze associations between metabolic parameters and damage markers. Peak torque declined significantly after 48 h (−25.64%), muscle stiffness was increased post (*p* = 0.004); CK peaked at 96 h (*p* < 0.001). For peak torque and CK, linear mixed models were fitted revealing significant contributions of △VO_2_% (*p* = 0.04) and △VCO_2_% (*p* = 0.08) to peak torque. Fixed effects explained 30.6% of the variance. Higher oxygen uptake (△VO_2_rec%) during rest intervals predicted lower CK elevations (*p* = 0.04). SmO₂ decreased by 13% within sets but had no significant effects on EIMD. Our findings indicate metabolic factors significantly contribute to EIMD in high‐velocity eccentric protocols. Greater aerobic demands within sets were associated with greater force deficits, whereas greater oxygen uptake during rest mitigated creatine kinase levels. Enhancing aerobic capacity and fatigue resistance could mitigate muscle damage and improve recovery trajectories in similarly demanding training contexts.

## 1 INTRODUCTION

Exercise‐induced muscle damage (EIMD) has traditionally been attributed to mechanical factors during eccentric exercise, leading to z‐line disruption and membrane damage (Proske & Allen, 2005). The accompanying symptoms of delayed‐onset muscle soreness (DOMS) cover a wide range of physiological mechanisms including enzyme leakage, force loss, reduced range of motion, and perceived muscle pain (Markus et al., 2021). However, significant increases in indirect damage markers such as creatine kinase (CK) have also been observed following endurance events (Overgaard et al., 2002; Pokora et al., 2014) and purely concentric protocols (Chen et al., 2000; Haralambie & Senser, 1980). These findings challenge the notion that EIMD is solely mechanically induced and suggest a metabolic contribution to muscle damage (Tee et al., 2007). While physiological processes like excessive Ca^2+^ accumulation (Gissel, 2005) or pro‐inflammatory responses (Suzuki et al., 2020) are associated with metabolic muscle damage, the specifics of these involvements remain poorly understood.

Beyond laboratory settings, overlapping effects of mechanical and metabolic factors in muscle damage also appear to be relevant in real‐world sports settings. Several sports have shown that the number of muscle injuries increases in the presence of muscle fatigue (Ekstrand et al., 2009; Gabbett, 2000; Jackson et al., 2013), indicating a possible overlap of fatiguing and damaging processes. The hamstrings are the most susceptible muscle group to injuries and the primary injury mechanism is the excessive stretching under load during the late swing of the running cycle in a fatigued state (Danielsson et al., 2020). Accordingly, the contraction velocity as well as the metabolic situation of the muscle during damaging exercise appear to be detrimental in the event of muscle injuries.

Early research on metabolic components of EIMD can be traced back to Abbott and Bigland (1953), who investigated the metabolic load during “negative work” using an eccentric cycling ergometer. They observed that the overall energy expenditure during eccentric contractions was lower compared to concentric contractions. This led to the assumption that the metabolic demand in eccentric exercise might be too low to contribute significantly to muscle damage (Abbott & Bigland, 1953). Subsequent studies confirmed that endurance‐based eccentric protocols elicit lower metabolic responses than concentric protocols (Isner‐Horobeti et al., 2013). This reduced metabolic cost is thought to result from mechanical cross‐bridge decoupling and the role of titin in passive lengthening contractions (Herzog, 2014). Few studies have specifically examined the metabolic response to eccentric resistance training, consistently reporting lower metabolic costs compared to concentric protocols (Fischer et al., 2021; Vallejo et al., 2006). Nevertheless, significant increases in oxygen uptake (VO_2_), carbon dioxide production (VCO_2_), and ventilation have been observed during maximal eccentric exercise (Paulus et al., 2019). The results of Paulus et al. (2019) indicate that under high‐intensity conditions, the metabolic demand of eccentric contractions may be higher than previously assumed, potentially contributing to muscle damage through mechanisms such as oxidative stress or energy depletion. Therefore, the role of metabolic stress in EIMD warrants further investigation, particularly in high‐velocity eccentric exercise settings.

At the muscular level, near‐infrared spectroscopy (NIRS) has evolved as a non‐invasive method to assess muscle oxygen saturation (SmO_2_) (Ferrari et al., 2011). SmO_2_ reflects the balance of oxygen delivery and consumption in the muscle tissue (Feldmann et al., 2019). During heavy resistance training, SmO_2_ typically decreases due to increased intramuscular pressures that restrict blood flow to the contracting muscle (Miranda‐Fuentes et al., 2021; Muthalib et al., 2010). This phenomenon is particularly relevant in eccentric exercise, where high force production and increased intramuscular pressures can lead to localized ischemia. Research indicates that such ischemic conditions not only reduce oxygen availability but also alter muscle oxygenation kinetics following EIMD (Caldwell et al., 2016; Davies et al., 2008), possibly contributing to the symptoms of DOMS. For example, decreases in tissue oxygenation index of up to 15% during a 10‐set eccentric training protocol for the elbow flexors have been reported. Similarly, significant decreases in muscle oxygenation have been reported in the m. vastus lateralis during continuous eccentric exercise until exhaustion (Denis et al., 2011; Szczyglowski et al., 2017). These findings suggest that the repetitive cycles of oxygen depletion and reoxygenation could induce oxidative stress, potentially contributing to muscle damage by the accumulation of metabolic by‐products.

In summary, although mechanical strain is regarded as the principal cause of EIMD, emerging evidence underscores the potential for metabolic stress—particularly in the context of high‐intensity eccentric contractions—to exacerbate muscle damage through processes such as ischemia–reperfusion or oxidative stress. Prior studies showed lower metabolic responses in eccentric versus concentric exercise primarily focused on elderly or untrained populations and moderate intensities, leaving high‐velocity eccentric protocols in trained populations largely unexplored. This gap suggests that we may be underestimating how systemic (VO_2_, VCO_2_) and peripheral (SmO₂) metabolic demands interact with mechanical factors to influence the extent of EIMD. Therefore, we hypothesized that greater metabolic stress during high‐velocity eccentric contractions elicits a more pronounced damage response. Understanding these metabolic underpinnings could refine our perspective on EIMD and inform preventative strategies for fatigue‐related overuse injuries in both athletic and clinical settings.

## 2 METHODS

### 2.1 Study Design & Intervention

The present study was approved by the local ethics committee and conducted following the ethical standards set by the Declaration of Helsinki. Further, it was pre‐registered at the German register for clinical trials (DRKS00031644).

A week before the intervention, participants attended a familiarization session including the eccentric and isometric contraction modes on the isokinetic dynamometer (ISOMED 2000, D. & R. Ferstl GmbH, Hemau, Germany). On the same day, anthropometric data were collected using a 3D body scanner (Scaneca GmbH, Berlin, Germany) and an incremental ramp test on a stationary bike (SRM ERGO, SRM GmbH, Jülich, Germany) was performed to assess the participants' VO_2_max.

**FIGURE 1:**
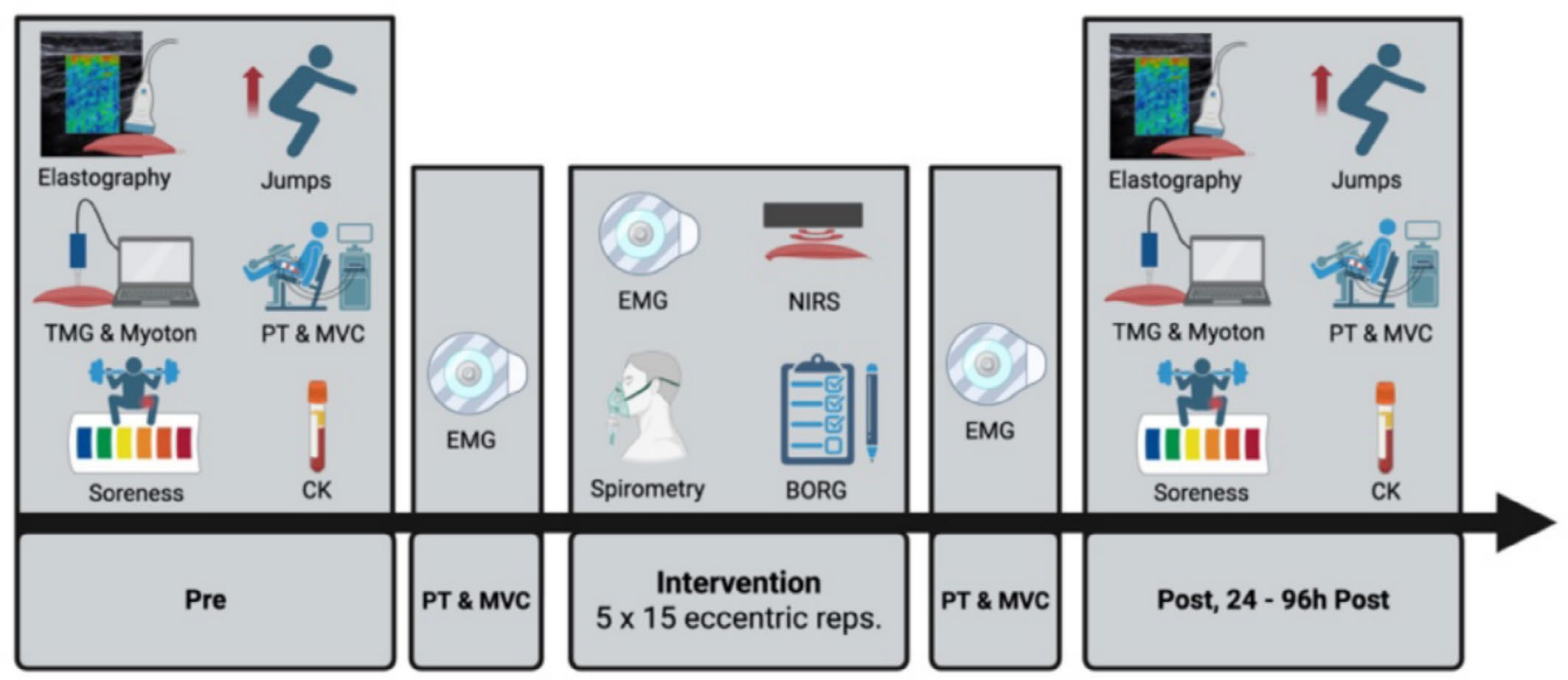
Experimental setup of the intervention. CK, creatine kinase; MVC, maximal voluntary contraction; NIRS, near‐infrared spectroscopy; PT, peak torque. This image was created with BioRender.

The intervention protocol consisted of five sets of 15 maximal eccentric hamstring curls performed in a seated position with one‐minute rest intervals between sets. Participants were seated with an 85° trunk flexion; the knee joint range of motion was individualized based on the anthropometric characteristics in a range of 0° to 100° knee flexion. Further, all participants were tightly strapped to the dynamometer, and the axis of rotation was aligned to the lateral femoral condyle, followed by gravitational moment correction (Zhang et al., 2021). The eccentric contraction velocity was set to 210°/s and the passive concentric part of the movement was set to 50°/s (Schwiete et al., 2023). Laboratory room temperature was maintained at 22.96 **±** 1.29C° throughout the intervention. Before, immediately after, and on the consecutive 4 days, several damage markers were collected from all participants. During the protocol, NIRS and EMG sensors were placed on the muscle belly of the m. biceps femoris, and a metabolic cart was used to assess oxygen consumption (see Figure 1). At the end of each set, participants were asked to rate their perceived exertion using the BORG scale.

### 2.2 Participants

An a priori power analysis using G*Power 3.1 (University Duesseldorf, Germany). Determined a sample size of 26 participants was necessary for a power of 0.80, with an effect size of *f* = 0.25 and an *α* = 0.05, based on the effects of a previous study in our lab (Schwiete et al., 2023). To account for dropouts 32 male trained participants were recruited for this study, of which 30 were used for the final data analysis. Only male participants were included as sex has been reported to influence EIMD (Markus et al., 2021). Inclusion criteria were that the participants had to be between 18 and 35 years of age, be free of injuries in the lower extremities, take no regular medications, and train at least twice per week. Participants with lower body injuries, regular medication, and less than two training sessions per week were excluded from study participation. The anthropometric characteristics and training volume of the study population are presented in Table 1. All participants gave their informed consent before they were included in the research.

**TABLE 1:** Characteristics of the study population. Data are in means ± SD.

Parameter	*N* = 30
Age	25.4 ± 2.8 (years)
Weight	81.5 ± 8.6 (kg)
Height	180.6 ± 7.4 (cm)
Basal metabolic rate	1916.2 ± 131.5 (kcal)
Resistance training/week	2.2 ± 2.5 (h)
Endurance training/week	1.1 ± 1.6 (h)
Recreational sports/week	1.5 ± 2.1 (h)
VO_2_max	36.2 ± 4.6 (mL/kg/min)

### 2.3 Study parameters

All study parameters were collected during the damage protocol.

#### 2.3.1 Eccentric peak torque and MVC

Participants performed a general five‐minute warm‐up on a stationary bike at 100 watts (60–70 rounds per minute), followed by two eccentric warm‐up sets on the isokinetic dynamometer. Maximal voluntary contraction (MVC) was measured at 90° knee flexion (Oakley et al., 2013) through a one‐repetition, five‐second maximal isometric hold. After a 3‐min rest interval, eccentric peak torque was assessed during one set of five maximal eccentric contractions with the contraction velocity of the intervention (210°/s eccentric). Both MVC and eccentric peak torque were assessed before and immediately after the intervention. Total work was recorded by the isokinetic dynamometer throughout the repetitions of the damage protocol and analyzed.

#### 2.3.2 EMG

The Delsys Trigno Wireless EMG System (Delsys Incorporated, Natick, USA) with a sampling frequency of 2000 Hz was used to record muscle activity during the intervention. The sensor was placed on the muscle belly of the m. biceps femoris at 50% of its length. Before the measurements, the region of interest was dry shaved and then cleaned with abrasive paste (Yaserifar & Souza Oliveira, 2022) according to the SENIAM guidelines. All data were analyzed with the EMGworks analysis software.

A median frequency analysis of the five‐second MVC before and after the intervention was performed to investigate the participants' muscular fatigue. Before the data analysis, an analog bandpass filter of 20–450 Hz was used, and EMG signals were mean‐centered and zero‐padded to the window length.

#### 2.3.3 Near‐infrared spectroscopy

Real‐time muscle oxygenation (SmO_2_) of the m. biceps femoris was monitored using the IDIAG moxy (Idiag GmbH) NIRS sensor with a 2 Hz sampling frequency and 15 mm sampling depth. Subcutaneous fat thickness at the sensor location was assessed via ultrasound (Acuson Redwood, Siemens, Germany), with an average thickness of 8.32 ± 3.7 mm (max.: 12.85 mm). The sensor was positioned below the EMG sensor on the muscle belly and fixated with a sleeve and tape to prevent light interference and slipping of the device. The start and end of the respective sets were marked and these data points were used for further data analysis. In addition, relative SmO_2_ changes within individual sets (△SmO_2_%) and rest intervals (△SmO_2_rec%) were analyzed (Gómez‐Carmona et al., 2020).

#### 2.3.4 Spirometry

Pulmonary gas exchange and ventilation were recorded breath‐by‐breath using the Cosmed Quark CPET (Cosmed GmbH, Germany), calibrated according to the manufacturer's recommendation. Markers were set in the Cosmed software at the start and end of each respective set. A five‐second average was used for the data analysis for each timepoint. Oxygen uptake (VO_2_) and carbon dioxide production (VCO₂) were analyzed to investigate the systemic metabolic response during the training intervention. Change scores were calculated regarding the relative changes during the sets (△VO_2_%, △VCO_2_%) and resting intervals (△VO_2_rec%, △VCO_2_rec%).

#### 2.3.5 Perceived exertion

At the end of each set, participants reported their perceived exertion using the BORG scale ranging from 6 to 20, where 7 represented “extremely easy” and 19 represented “extremely exhausting.”

#### 2.3.6 Creatine kinase

Capillary blood was collected and immediately centrifuged (Universal 320 R, Andreas Hettich GmbH, Tufflingen, Germany) for 10 min at 3000 rounds per minute. Afterwards, 10 μL of blood plasma was collected with a reusable pipette (Eppendorf Research Plus, Eppendorf, Hamburg, Germany) and analyzed in a point‐of‐care‐testing system (DRI‐CHEM Analyzer FDC NX500, Fujifilm Europe, Duesseldorf, Germany). As the testing system is only capable of detecting CK levels of up to 2000 U/L, post‐exercise samples were manually diluted in the form of a dilution series (1:2; 1:4; 1:8; 1:16; 1:32; 1:64), using a concentrated 0.9% sodium chloride solution. The value of the lowest dilution was used for the final data analysis.

#### 2.3.7 Shear‐wave elastography

**FIGURE 2:**
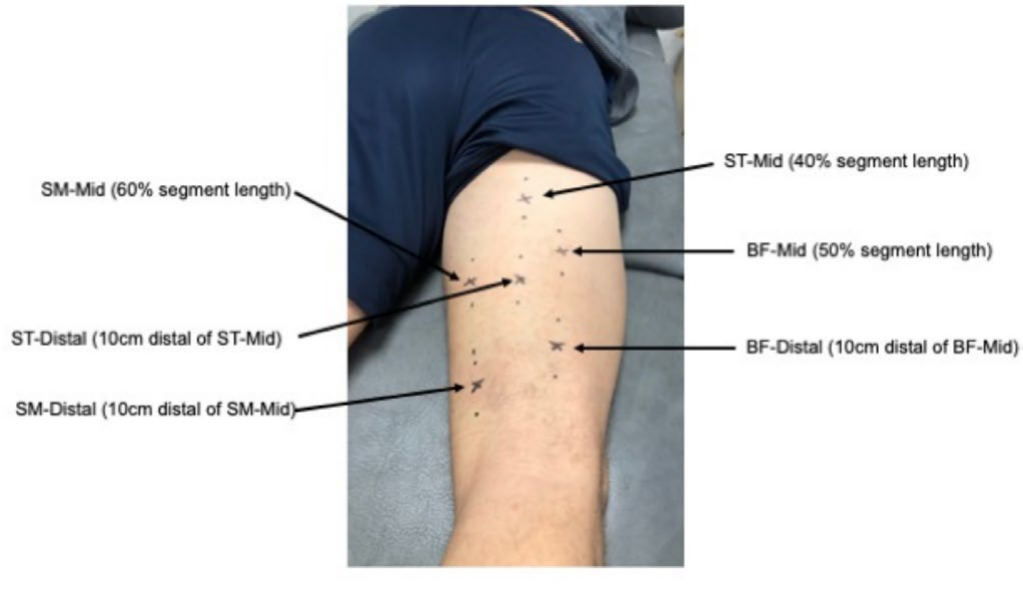
Measurement locations for the elastography and tensiomyography assessments. BF, m. biceps femoris; SM, m. semimembranosus; ST, m. semitendinosus. The participant consented to the publication of this image.

Shear‐wave elastography allows the quantification of elastic and mechanical properties of tissues, for example, muscle stiffness (Ryu & Jeong, 2017). Information about muscle stiffness can help to better understand muscle injuries as increased muscle stiffness is associated with overuse injuries (Mueller‐Wohlfahrt et al., 2013). An ACUSON Redwood (Siemens Healthineers, Erlangen, Germany) was used, and all measurements were conducted by the same experienced investigator. A 10L4 linear array probe (50 mm width) was placed longitudinally on six different measurement sites. The sites were adapted from Kawama et al. (2022) and included one measurement point on each muscle (50% of biceps femoris length, 40% of semitendinosus length, 60% of semimembranosus length). An additional site was added 10 cm distal to the respective measurement site, resulting in a total of six measurement locations (Figure 2). Anatomical landmarks, imaged by the ultrasound system, were used as reference marks for the region of interest, which was 6 × 10 mm and placed beneath the upper fascial sheath. Within the region of interest, we evaluated elastography values in a measuring circle (10 mm diameter). Three consecutive measurements were performed at each measurement site, and mean scores were calculated. A main score, consisting of the values of all measurement locations, was automatically calculated by the ultrasound device, and this main score was used for the data analysis to get an overview of the stiffness changes in the whole hamstring group.

#### 2.3.8 Tensiomyography and myoton

TMG measurements (TMG‐BMC Ltd., Ljubljana, Slovenia) were conducted at the same sites as the elastography measurements, with the participants lying in a prone position. The ankles were placed on the TMG cushion for lower leg measurements to generate a knee flexion of 5° (Wilson et al., 2019). Two electrodes (self‐adhesive, dura‐stick, 50 × 50 mm) with an inter‐electrode distance of 5 cm were attached to the skin (Piqueras‐Sanchiz et al., 2020) and the location was marked with a water‐resistant marker. Per site, three measurements were performed at 100 mA with a 30‐second resting interval in between. In line with our elastography approach, a main score was calculated for radial displacement (*D*_m_) for all measurement sites, and this main score was used for the final data analysis.

Myoton (Myoton Ltd., Tallinn, Estonia) was used in multiscan mode with three consecutive impulses to evaluate dynamic muscle stiffness (S, N/m) (Schneebeli et al., 2020). Myoton measures the radial deformation of the tissue in response to the applied force from the sensor (0.4n), with reliable and valid results (McGowen et al., 2023).

#### 2.3.9 CMJ, SJ, and DJ

Jumping performance was assessed using an opto‐electronic measurement system (OptojumpRX10, MicroGate, Bolzano, Italy). All jumps were introduced to the participants regarding correct execution on two occasions before the start of the intervention. All jumps were performed with the participants' hands on their hips. Three jumps were executed with at least 30 s rest per jump. For SJs, participants were instructed to hold a 90° squat position for a minimum of 1 s. DJs were performed from a 30 cm box; ground contact time had to be shorter than 200 ms for the jumps to be included in the data analysis.

#### 2.3.10 Muscle soreness

Muscle soreness was assessed using the visual analog scale. The scale was 100 mm long (3.94 inches) and ranged from “no pain at all” to “worst pain imaginable” (Heller et al., 2016). All participants were instructed about the proper utilization of the scale.

## 3 STATISTICAL ANALYSIS

For statistical analyses, the IBM SPSS Statistics software (version 28.0), Jamovi (version 16.44), and Graphpad Prism (Version 10.3) were utilized. Linear mixed models were used to analyze the effects of the metabolic changes during the training on the damage markers. To account for interindividual differences, the participants' baseline values were included as a random effect. Time as well as the change scores of SmO_2_ (△SmO_2_%, △SmO_2_rec%), VO_2_ (△VO_2_%, △VO_2_rec%), and VCO₂(△VCO_2_%, △VCO_2_rec%) were included as fixed effects. Model fits were evaluated via BIC and AIC and covariates were added or removed accordingly. One‐way repeated measures ANOVAs were used to identify the changes in all dependent parameters. All post hoc comparisons were conducted using the Bonferroni correction. EMG values during the pre‐ and post‐exercise MVC were compared using one‐sample *t*‐tests.

Preceding, all assumption tests for the statistical methods were carried out. All data were checked for normality (Shapiro–Wilk test) and outliers. In addition, Levene's test for an inhomogeneous variance for one‐way ANOVA was employed. Greenhouse–Geisser correction was applied if Mauchly's test indicated a violation of sphericity. All tests were based on a 5% level of significance. Data are presented in means ± SD for the ANOVAs, and as median for the EMG values.

## 4 RESULTS

### 4.1 Metabolic contributions to EIMD

Linear mixed models showed significant metabolic contributions to the damage markers CK and peak torque. For CK, a mixed model was fitted with the factors time, △VO_2_rec%, △VCO₂rec%, △SmO_2_rec% as fixed effects and random intercepts by participants. Model fit indicators showed that fixed effects explained 14.8% of the variance (marginal *R* ^2^ = 0.148), including random effects, bringing the total explained variance to 39.2% (conditional *R* ^2^ = 0.392). The model revealed time (*p* < 0.001) and △VO_2_rec% as significant predictors (*p* = 0.04). △VCO₂rec% (*p* = 0.08) and △SmO_2_rec% (*p* = 0.98) did not contribute significantly. △VO_2_rec% showed a negative association with the CK response (estimate = −387.67, SE = 181.2). This suggests that a higher oxygen uptake during the resting intervals predicts lower CK concentrations.

For peak torque, a mixed model was fitted with the factors time, △VO_2_%, △VCO₂%, △SmO_2_% as fixed effects and random intercepts by participants. Fixed effects explained 30.6% of the variance (marginal *R* ^2^ = 0.306) with random effects explaining a total variance of 74.6% (conditional *R* ^2^ = 0.746). There were significant main effects for time (*p* < 0.001), with △VO_2_% (*p* = 0.04) and △VCO₂% (*p* = 0.03) emerging as additional predictors.

### 4.2 Effect on damage markers

**FIGURE 3:**
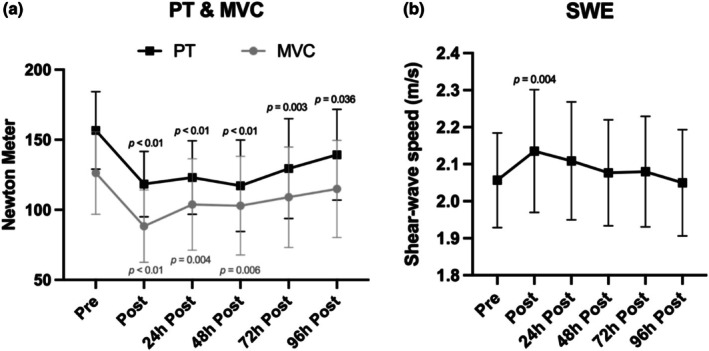
a+b: (a) PT and MVC changes throughout the study. (b) Development of muscle stiffness during the study. MVC, maximum voluntary contraction; PT, peak torque; SWE, shear‐wave elastography.

There were significant losses in eccentric peak torque (*p* < 0.001, *η* ^2^ = 0.44) and MVC (*p* < 0.001, *η* ^2^ = 0.37) of 25.64% and 17.81% after 48 h, respectively (Figure 3). Concomitantly, muscle soreness (*p* < 0.001, *η* ^2^ = 0.38) and CK (*p* < 0.001, *η* ^2^ = 0.17) increased significantly, with soreness peaking after 48 h but CK elevating until 96 h post‐exercise. Jumping performance significantly decreased in all jumps immediately after the damage protocol (CMJ = −10.7%, SJ = −6.7%, DJ = −6.8%). A significant time effect was found for elastography (*p* < 0.001, *η* ^2^ = 0.18). Shear‐wave speed in the hamstrings increased from 2.06 ± 0.13 m/s at baseline to 2.13 ± 0.17 m/s immediately post (*p* = 0.004) before decreasing again. For muscle contractility, there were significant main effects for *D*_m_ (*p* < 0.001, *η* ^2^ = 0.21). There were no effects for passive muscle stiffness measured by Myoton (*p* = 0.1, *η* ^2^ = 0.07).

### 4.3 Effect on metabolism

Subcutaneous fat did not significantly affect muscle oxygenation at rest *F*(1,27) = 0.10, *p* = 0.92 and only explained 3.7% of the variance (adjusted *R* ^2^ = −0.037). The damage protocol elicited significant changes in SmO_2_ over time (*p* < 0.001, *η* ^2^ = 0.28). While not statistically significant, SmO_2_ increased throughout the damage protocol from 71.53 ± 12.27% at the onset of the first repetition to 76.6 ± 20.16% at the start of the last set. Within the sets, SmO_2_ decreased by an average of 13.03 ± 19.47% with the largest changes in the fourth set. During the resting intervals, SmO_2_ recovered by 25.88 ± 43.24% on average.

There were significant time effects for the spirometry parameters (VO_2_ = *p* < 0.001, partial *η* ^2^ = 0.44; VCO₂ = *p* < 0.001, partial *η* ^2^ = 0.47). For VO_2_ and VCO₂, all time points differed significantly from the pre‐value. Within sets, VO_2_ and VCO_2_ increased by 47.78 ± 63.41% and 49.8 ± 60.19% on average, respectively.

There was a significant time effect for total work (*p* < 0.001, partial *η* ^2^ = 0.52) as it decreased from 1560.9 **±** 381.7 to 1260.1 **±** 332.2 J from the first to the last set, respectively. Inversely, the BORG score showed a significant time effect (*p* < 0.001, partial *η* ^2^ = 0.73) with increases in perceived exertion from 13.8 **±** 2.01 to 17.7 **±** 1.49 throughout the sets. Regarding median frequency, there was a significant decrease after the damage protocol (*p* < 0.001) from 75.9 **±** 13.9 to 73.7 **±** 16.4 Hz. Taken together, these results prove that the applied training protocol induced significant muscle fatigue in the hamstring muscles.

## 5 DISCUSSION

As expected, our protocol led to significant alterations in all primary damage markers. From a neuromuscular perspective, eccentric peak torque and MVC showed the largest force decreases after 48 h (−25.64% peak torque) indicating moderate muscle damage based on the categorization of Paulsen et al. (2012). In addition, muscle soreness peaked after 48 hours (3.27 ± 4.74 to 30.34 ± 20.72 mm). Our results also indicate that complete recovery for muscle soreness and CK was not reached even after 96 hours as both were still significantly elevated.

### 5.1 Metabolic contributions to EIMD

Overall, our findings highlight a significant metabolic contribution to exercise‐induced muscle damage (EIMD), particularly affecting post‐exercise peak torque and CK responses. Our model demonstrated that oxygen uptake and carbon dioxide production during exercise significantly influenced the peak torque decline at 24 h post‐exercise. Specifically, ΔVO2% (*p* = 0.04) showed a negative association with post‐exercise peak torque, while ΔVCO_2_% (*p* = 0.03) was positively correlated. Although both parameters typically increase concurrently, their effects on peak torque were contradictory. This discrepancy may be explained by differences in the timing and rate of metabolic responses during the loading protocol, potentially leading to divergent impacts on muscle function.

Notably, a higher aerobic energy demand (ΔVO2%) was associated with greater force decrements, which challenges the assumption that aerobic metabolism mitigates muscle damage. Possible explanations include an increased mechanical workload, impaired mitochondrial efficiency, or a greater reliance on fatigue‐prone slow‐twitch fibers under eccentric loading. Additionally, elevated ventilatory demand and central fatigue could have contributed to neuromuscular impairments. Further analyses, including mechanical workload and local muscle oxygen saturation (NIRS), may help clarify these relationships.

Overall, the results of our study show significant metabolic contributions to the induced EIMD. Metabolic effects were found affecting the post‐exercise peak torque and CK response. Our model revealed that oxygen uptake (△VO_2_%) and carbon dioxide production (△VCO₂%) during exercise significantly affected the amount of peak torque decrease at 24 h after.

Although △VO_2_% (*p* = 0.04) revealed a negative association, conversely △VCO₂% (*p* = 0.03) was positively related to peak torque. These seemingly contradictory results could be due to differences in timing and slope of the relative increases during the set, possibly leading to deviating effects on peak torque. Regarding the contributions of △VO_2_%, our model indicates that a higher demand for aerobic energy during the damage protocol led to larger force decrements.

In line with this, our CK model showed a negative relationship with △VO_2_rec% (*p* = 0.04), meaning that a higher recovery of oxygen uptake during the resting intervals predicts lower CK concentrations. These results support the work of (Hody et al., 2013) who reported significant correlations between force decreases during eccentric exercise and the post‐exercise CK response. The authors concluded that muscle fatigue, defined as force declines, could predict the magnitude of EIMD. While our models only explained 30.6% (peak torque) and 14.8% (CK) of the variance in our population, greater VO_2_ alterations might mitigate the subsequent damage response through different mechanisms. Higher oxidative metabolism during sets is critical as it helps the muscle to clear metabolic by‐products like hydrogen ions or inorganic phosphate. These by‐products are believed to play a central role in the metabolic contributions to EIMD, as they impair Ca^2+^‐release and reuptake from the sarcoplasmic reticulum (Allen et al., 2008). Research on rats has shown that intense, prolonged exercise can lead to a 10‐fold increase in Ca^2+^‐concentrations, which persist even after the exercise. Excessive intracellular Ca^2+^‐concentrations possibly contribute to EIMD by activating Ca^2+^‐dependent phospholipases and proteases, leading to a loss in cross‐bridge function and membrane integrity (Gissel, 2005; Tee et al., 2007). Next to these direct effects, higher metabolic stress could also enhance EIMD indirectly by increasing the vulnerability of the muscle to damage (Tee et al., 2007). We know, for example, that fatigued muscles need to be further stretched to absorb the same amount of applied force compared to non‐fatigued muscles, thereby increasing muscle injury risk (Mair et al., 1996). Accordingly, better fatigue resistance might protect the muscle from greater amounts of muscle damage, which is supported by the results of our study.

### 5.2 Metabolic changes during eccentric exercise

#### 5.2.1 Systemic level

While data usually show rather small metabolic responses during eccentric compared to concentric resistance training (6.7 **±** 1.3 vs. 10.0 **±** 1.6 mL/kg/min) (Fischer et al., 2021), spirometry markers in our study changed significantly throughout the training protocol. On average, VO_2_ and VCO_2_ increased by 47.78 **±** 63.41% and 49.8 **±** 60.19% within sets. From the first to the last repetition of the whole training, VO_2_ and VCO_2_ even increased by 79.47 **±** 52.08% and 84.38 **±** 55.60%. A possible explanation for the more pronounced metabolic responses in our study compared to the studies of (Fischer et al., 2021) and (Vallejo et al., 2006) could be the training status (elderly/untrained vs. trained participants) of our study population combined with a higher eccentric contraction velocity (210°/s).

Trained individuals usually have better muscle recruitment, greater force production and can endure higher levels of perceived exertion (Gabriel et al., 2006). In addition, contraction velocity is known to affect VO_2_ consumption (Buitrago et al., 2013; Hunter et al., 2003; Mazzetti et al., 2007) in several ways. At faster contraction rates ATP consumption increases, resulting in higher overall metabolic stress for the muscle (Buitrago et al., 2012). Further, muscle contractions at a faster velocity are heavily dependent on higher‐threshold motor nerves and fast‐twitch muscle fibers, which are more energy‐consuming than their slow‐twitch counterparts (Dugan & Frontera, 2000; Müller‐Wohlfahrt et al., 2018). This enhanced metabolic demand of fast‐twitching fibers may also account for the increased injury risk in a fatigued state (Tee et al., 2007). Consistent with this, our results show the greatest changes in systemic metabolism towards the end of the protocol, when muscle fatigue was greatest, as demonstrated by the decrease in muscle work (−19.8%), median frequency (75.9–73.7 Hz) and increases in perceived exertion (13.8–17.7 BORG).

#### 5.2.2 Muscular level

On the muscular level, SmO_2_ decreased by 13% within sets, which is in line with the results of Muthalib et al. (2010), who had similar decreases in tissue oxygenation index throughout their 10‐set eccentric biceps training. These results demonstrate that EIMD changes the muscle oxygenation relationship towards an increased utilization in the muscle with a concomitant supply deficit in oxygen. Maximal eccentric contractions are known to be associated with high intramuscular pressure due to greater torque levels that can lead to microcirculatory dysfunctions (Denis et al., 2011; Lauver et al., 2020). In our study, muscle oxygenation was highly increased during the resting intervals, with SmO_2_ recovering by over 25% beyond baseline. These compensatory changes in O₂ delivery are necessary to even out the microvascular pressures during the exercise to maintain O₂ kinetics in the working muscles (Davies et al., 2008; Lauver et al., 2020). Our results confirm these fast‐acting vasodilatory counter‐processes as SmO_2_ levels were elevated at the onset of the last set compared to the first set (71.53 ± 12.27% vs. 76.6 ± 20.16%). These changes in oxygen kinetics are important to counterbalance acute microvascular alterations and help maintain muscle function. However, this constant exposure to ischemia and reperfusion is also associated with high levels of oxidative stress, which has been reported as one of the major metabolic contributors to muscle damage (Su et al., 2010). Ischemia in the muscle is known to trigger the release of free radical oxygen species (Sjödin et al., 1990) and lowers ATP levels significantly (Tupling et al., 2001), which results in an increased influx of calcium. Ultimately, this calcium influx can lead to the activation of Ca^2+^‐dependent phospholipases and proteases, which can damage the muscle cytoskeleton (Behringer et al., 2014).

## 6 CONCLUSION

The findings of our study indicate that although mechanical strain remains the primary driver of EIMD following high‐velocity eccentric training, metabolic factors also play a measurable role. We observed significant force decrements and elevated muscle soreness, muscle stiffness, and CK levels, suggesting moderate muscle damage. At the same time, higher aerobic demands during the sets were associated with larger peak torque reductions, whereas enhanced oxygen uptake recovery during rest intervals predicted smaller increases in CK. While not being statistically significant, muscle oxygenation measured by NIRS showed a pronounced decrease within sets and rapid recovery afterward, without significantly affecting EIMD markers. These results highlight that, in trained individuals performing maximal eccentric contractions at high velocities, both mechanical and metabolic factors contribute to the spectrum of muscle damage. Appreciating this combined influence may guide more effective training strategies and recovery protocols, potentially mitigating injury risk and optimizing performance adaptations.

## 7 LIMITATIONS

Several limitations should be considered when interpreting our findings. First, the study population consisted solely of male, trained participants, limiting the generalizability of the results to female or untrained individuals. Second, although multiple damage markers were measured, more direct assessments of intracellular muscle damage mechanisms—such as histological analyses—were not performed. Finally, while our protocol successfully induced fatigue and damage concurrently, a configuration emphasizing more repetitions and volume could have elicited a stronger metabolic response, potentially altering the observed balance between mechanical and metabolic stress. Future research should focus on expanding participant demographics and employing more direct muscle analyses.

## AUTHOR CONTRIBUTIONS

Carsten Schwiete wrote the first draft of the manuscript. Carsten Schwiete and Michael Behringer were responsible for planning the study and for the study design. Carsten Schwiete, Joachim Mester, Patrick Wahl, Holger Broich, and Michael Behringer revised the manuscript. The corresponding author attests that all listed authors meet authors hip criteria and that no others meeting the criteria have been omitted.

## FUNDING INFORMATION

No fundings were received for this study.

## CONFLICT OF INTEREST STATEMENT

The authors declare no conflicts of interest.

## ETHICS STATEMENT

This study was reviewed and approved by the Ethics Committee Department 05, Goethe University (no.; 2023‐35) and was pregistered at the German register for clinical trials DRKS00031644. This study was performed in line with the principles of the Declaration of Helsinki.

## CONSENT

The participants gave informed written consent to the main study and to receive invitations to sub‐studies.

## Data Availability

The raw data supporting the conclusions of this article will be made available by the authors, without undue reservation.
